# The Anatomy, Physiology, Pathology, and Remedial Treatment of the Fifth Pair of Nerves

**Published:** 1860-12

**Authors:** J. H. M’Quillen


### THE ANATOMY, PHYSIOLOGY, PATHOLOGY, AND REMEDIAL TREATMENT OF THE FIFTH PAIR OF NERVES.

BY J. H. M'QUILLEN, D. D. S.

(Concluded.)

SATISFIED that no pathological doctrines can command confidence which are not founded upon accurate views of the structure and functions of a diseased part, thus far, in the presentation of this subject, attention has only been directed to the anatomy and physiology of the fifth pair; having dwelt sufficiently on those points to give at least a general view of their structure and functions, as a natural sequence the pathology of this nerve, and the sympathetic derangements frequently induced in other parts of the organism under such circumstances, very properly command attention.

During the period of dentition the fifth pair is always more or less affected, and in excitable states of the nervous centers the irritation of it consequent upon the pressure of the teeth often gives rise to convulsions and other disturbances of the general organism, so common to that period of childhood. Indeed, the diseases pertaining to this period may be dependent, and in many instances are entirely so, upon the local irritation attending the process being transmitted through either the *cerebro-spinal system of nerves*, producing convulsive diseases in the motor apparatus ; or through the *sympathetic nerve*, causing derangement of the alimentary or pulmonary organs.

In illustration of this, two interesting cases came under my notice ; one of which, a little girl aged two and a half years, was seized, one morning in the fall of the year, with slight convulsions, which increased in number and severity toward the close of day. Taking into consideration the season of the year, the location of the residence in a region which was formerly regarded as eminently malarious, and the fact that intermittent fever was then quite prevalent, the family physician and myself were disposed to regard it as a case of fever and ague ; an examination of the mouth, however, was made, and the gum over the right and left lower second molars being found quite tumid, free crucial incisions of it were made with the lancet. Immediate relief was afforded, and no return of the convulsions supervened.

In the second case, a little boy, about the same age, was attacked with spasmodic croup, an affection which consists in a sudden choking fit, caused by spasm of the muscles that close the glottis, and differing from croup in the fact that there is no fever nor any morbid appearances about the throat; the disorder being purely functional, and having its origin in irritation of a distant part, which derives its nerves from the same region of the cerebro-spinal centers with the larynx. That such was the case in this instance, was proved by the fact that soon after lancing the gums over the deciduous molars, the difficulty disappeared.

The advice that Dr. J. K. Mitchell endeavored to impress upon the minds of his students can not be too strongly advocated. It was, that when called to the side of a little sufferer, laboring under whatever affection it may—whether cerebral, thoracic, or abdominal—during the period of dentition, to immediately examine the mouth of the patient, and if there should be the slightest evidence of the eruption of a tooth, to employ the lancet at once, making, in the case of the molars, decided crucial incisions.

The affection most frequently presented by the fifth pair, occurring in whatever branch of the nerve it may, and whether originating at the periphery or center, can be classified under the general head of *neuralgia*—a term, it is true, often used in the most vague and indefinite manner by medical and dental practitioners, to conceal from the patient or friends their want of familiarity with the cause of the trouble which they are called upon to relieve. In support of this, numerous cases have presented themselves in which patients were subjected for weeks and months to the most varied constitutional treatment, by the medical practitioner, when all that was demanded was the removal of an exposed dental pulp, or possibly a tooth; and, on the other hand, many sound and valuable teeth have been sacrificed by the dental practitioner, when the source of trouble may have been in some other part of the nerve, or even in a distant organ. Frequently, it is true, the most experienced and accurate observers have been unable to discover the cause or satisfactorily account for the phenomena presented in this affection during the life of the patient; but, fortunately for science, in some instances the *post mortem* examination has revealed in the clearest manner the deep-seated cause of mischief.

In employing *neuralgia* as a generic term for the painful affections known as *tic douloureux*, and *odontalgia*, it is merely used as the verbal definition of a symptom—pain in a nerve. Of this pain there are different degrees of severity experienced, sometimes being moderate, and at others unendurable; the duration varying from intervals of a few seconds to a day, or every other day, or even a much longer interval of time. When severe, it usually comes on suddenly, with a sharp, darting or tearing character, and is either confined to one spot, or it may extend to all of the branches of one side of the head and face. In attempting to discover the cause of this very painful affection, the origin, distribution, and sympathetic connections of the nerve should be constantly borne in remembrance. Thus it may have a peripheral, a central, a reflex, or a general origin.

*Peripheral origin.*—Prolonged irritation of the terminal extremity of one of the branches, as in exposure of the dental pulp to variations of temperature, the contact of foreign bodies ; or in pressure upon the dental pulp, as during the period of primary or secondary dentition.

*Central origin* may be owing to pressure upon the nerve or one of its branches, from the growth of a tumor, or a spicula of bone, or the periosteal or osteal thickening of the osseous foramen through which the different branches of the nerve emerge from the cavity of the cranium.

*Reflex origin* may be due to the irritation in some distant organ exciting sympathetic pain in the fifth pair, or *vice versa*, and in the latter frequently with considerable disturbance of the function of the parts.

*General origin.*—Depressing influences of all kinds are especially likely to produce it; thus, mental depression, debilitating diseases, exposure to malaria or inclement weather, or sudden suppression of any of the important secretions, or of the catamenial discharge, are each and all prolific causes.

By some writers it is asserted that although considerable attention has been paid to the morbid anatomy of nerves affected with neuralgia, nothing has been made out in this direction which throws any light on the nature of the disorder. Dr. Cornochan, however, states that in three cases in which he exsected the second branch of the fifth pair of nerves, he found the exsected portion red, vascular, engorged, and considerably enlarged ; hence he regards vascular engorgement or inflammation, with some of its consequences, of the neurilemma alone, or of it and the nerve together, by whatever cause produced, as the condition which constitutes the pathological changes in the trunk.

Arising, as *neuralgia* does, from such a variety of causes, the diagnosis, though usually effected without much difficulty, is, as already stated, in some cases quite embarrassing. What increases the difficulty in making out these nervous pains is, that they may be produced by some source of irritation situated at a distance from the part in which the pain is felt. As a familiar illustration of this position, if the ulnar nerve is struck at the elbow, a peculiar tingling sensation is felt, not in the part struck, but in the sentient extremity of the same nerve distributed to the little finger. This example proves how necessary it is, when no cause of pain can be found in the part complained of by the patient, to look for the source of irritation in another portion of nerve, or in some distant organ.

In by far the vast majority of cases, neuralgia of the fifth pair has a peripheral origin, resulting from caries of the teeth, and denominated *odontalgia*. Of this there are three varieties : true, false, and sympathetic toothache ; and it is in diagnosing these varieties that the ability, judgment, and experience of the dentist is demonstrated. It is also a subject well worthy of the careful consideration of the medical practitioner. The peculiarities presented and the manner of diagnosing the different varieties are as follows :

*Odontitis.*—True toothache, or inflammation of the dental pulp, is demonstrated by the following evidence : pain, severe and constant, and usually confined to the diseased organ, but not unfrequently affecting other branches of the fifth pair distributed to the side of the head and face; the pressure of foreign substances, such as particles of food, the edge of an excavator, or the point of a probe, upon the exposed pulp, and exposure to sudden variations of temperature, either of air or fluids, increase the pain, which becomes more intense toward and during the night; added to this may be the visible exposure of the pulp, or, when that is not accurately defined, the oozing of blood or serum into the cavity of decay. In doubtful cases, if on packing a pledget of cotton into the cavity, after the caries has been removed, the pain is increased, and continues after the removal of the cotton, the pulp is exposed; but should it lessen in a few seconds and eventually cease, a thin covering of dentine will be found protecting the pulp.

*Periodontitis.*—False toothache, or inflammation of the periodental membrane of the fang, like inflammation in other parts of the body, is divisible into three stages, viz: simple vascular excitement, active congestion, and true inflammation. In the first stage, which is simply a hyperæmic condition of the capillary vessels of the membrane, the circulation through the part proceeding at the same time with unwonted velocity, there is merely a *slight amount of tenderness, which is increased by striking sharply on the affected tooth with the handle of an instrument.* A sense of relief is obtained by gradual and continued pressure upon the tooth, which, however, on being removed, is followed by a recurrence of the tenderness. Should the inflammatory action not cease here, but proceed, *active congestion* will be established, the capillary vessels of the part becoming more and more distended, until farther distention is no longer possible, owing to the confined locality of the tissue; *the most excruciating pain is now experienced, and along with it intolerance of the slightest pressure. On this account, closure of the teeth is avoided, as it but aggravates the evil. Owing to the distention of the blood-vessels of the membrane, the tooth is forced slightly out of its socket, and thus feels longer and looser than its neighbors.*

The pain arising from odontitis or periodontitis is not always located in the seat of difficulty, but is frequently referred by the patient to a perfectly sound tooth, thus constituting sympathetic toothache ; for instance, it is not an unusual circumstance for patients to direct attention to a superior central incisor as the cause of excruciating suffering, which, on careful examination, will be found perfectly sound ; every argument to convince the patient that such is the case proves unavailing, however, until in the course of the examination the operator touches an exposed pulp in a canine, a bicuspid, a first or second molar, or a wisdom tooth, in the upper jaw, or possibly even in the lower.

The sympathetic irritation arising from odontitis or periodontitis, when extending beyond the dental organs to other parts of the fifth pair, may either be located in the face, simulating tic douloureux, or it may, as in the following case, mentioned by Lawrence, not only induce intense pain in the eye, but also bring on impaired vision, or amaurosis.

"*Amaurosis caused by a carious tooth.*—F. P., thirty years of age, possessing a good constitution, and enjoying good health, with the exception of pains in the head and limbs, which never lasted long, suddenly experienced, in the autumn of 1825, a violent pain shooting from the left temple to the eye and the side of the face ; he ascribed it to cold. This pain lasted several days, then lessened, and reappeared from time to time without being sufficiently severe to induce the patient to seek medical aid. In about two months it suddenly increased in intensity, occupying the eye particularly with a feeling as if it would pass out of the orbit. F. P. now discovered that he was blind with that eye, and applied to a neighboring physician, whose treatment, continued for two months, did no good. The pain, however, was no longer continual ; it assumed a periodical character, leaving the patient easy for some hours in the day. At the end of the following six months the pain increased, the cheek swelled ; some spoonsful of bloody matter were discharged by a spontaneous opening in the lower eyelid, after which the swelling subsided, and the pains nearly disappeared, although the blindness remained complete. The discharge was renewed from time to time during the following six months, and there was no great suffering. But in the autumn and winter (1826) the pain, particularly in the eye, became so violent, that F. P. came to Wilna in the beginning of 1827, determined to have the organ extirpated, if no other remedy could be found. Professor Galenzowski found the left eye totally insensible to light, with the pupil dilated, and no other visible alteration. The pain, not then so severe, consisted in violent occasional pricking or darting sensations in the left temple, and parts round the eye. There was discharge from the lower eyelid. The first molar tooth of the left side was carious; it had not caused much uneasiness; and the toothache, when it existed, had not coincided with the pains in the temple and eye. The professor determined on removing this tooth, and having done so, was surprised to see a foreign body at the extremity of the fang. When drawn out, it proved to be a small splinter of wood, about three lines in length, which had traversed the center of the tooth, and had probably been introduced in picking the teeth. A probe was passed from the socket into the antrum, from which a few drops of thin, purulent fluid escaped. The pain ceased almost entirely, and on the same evening the eye began to be sensible to light. Vision gradually improved, so that on the ninth day the patient could see as well with the left eye as with the right, after a blindness of thirteen months; on the eleventh day he left Wilna, to return to his family."

Numerous cases might be cited in which amaurosis had resulted from dental irritation, and been relieved by the extraction of the diseased teeth. Of these it will suffice to refer to one or two only. Mr. Tavers, for instance, "saw an incipient amaurosis distinctly arrested by the extraction of a diseased tooth, when the delay of a similar operation had occasioned gutta serena, or total blindness, on the opposite side, two years before." Dr. Watson states "that the son of a physician of his acquaintance, in London, became blind in one eye on two or three occasions, without obvious cause, and with no visible change in the organ, and the blindness on each occasion went off, apparently in consequence of the extraction of some teeth which had grown irregularly."

On different occasions, cases have come under my own notice in which the most excruciating pain in the eye has been complained of by patients, which unquestionably arose from dental irritation, as it passed off with proper treatment of the teeth. The only case of perverted function of the eye, however, that my attention has been directed to by a patient, was in the case of a gentleman, who stated that many years ago when under the hands of another operator, he had a right upper central incisor treated, which was very sensitive, and caused considerable pain in the right side of the face and eye; immediately after the operation, he found that he had an attack of strabismus—a difficulty which he labored under, to a slight degree, in childhood, but which had passed off entirely. The strabismus, accompanying the operation, continued for a few days only, when it gradually disappeared.

If, after a careful examination of the dental organs, no cause for the terrible neuralgic pains, known as *tic douloureux*, can be found in the peripheral extremities of the nerve, it must be referred to one of the many causes already alluded to. Of those cases having a central origin, it must be evident to all that the probabilities of a cure are very slight, on account of the impossibility of reaching them with remedial agencies. And it is in these cases where the suffering of the patient is most terrible, their quivering features and restless limbs indicating that the acute bodily pang is indeed hard to bear. The pain is usually restricted to one of the three branches, though sometimes two, and in others all of them are implicated. The second branch of the fifth pair is, perhaps, more frequently affected than either the first or third.

Apparently the most trivial causes are often sufficient to provoke or renew paroxysms of suffering in this affection.

Thus the necessary movements of the face in speaking or eating, a slight touch, a current of air upon the face, or a knock at the door, are sufficient to start it. Individuals whose powers have been broken by advancing years or debilitating diseases, and those who are pale and asthenic, are most apt to be victims. Derangement of the digestive organs frequently accompanies, and, in some instances, is the exciting cause of this difficulty. An interesting case has been reported by Dr. Suesserott, in which the patient had a number of teeth removed, and was subjected to a long-continued course of constitutional treatment without any benefit, until Dr. S. discovered that he was in the habit of swallowing his food, meats, etc., in large pieces, and without any attempt at mastication. On ascertaining this fact, he was directed to pay proper attention to the comminution of his food, and this was followed by the most happy results, the neuralgic affection of the face soon disappearing altogether.

In the treatment of this affection, the first indication, of course, is to remove the exciting cause. When this is dependent upon *odontitis*, the application of the arsenical paste to the exposed pulp and the removal of this on the following day, or, when due to *periodontitis*, the employment of leeches to the gum, and the administration of a mild cathartic, may prove all-sufficient. But if it has a more complicated origin, the endeavor must be made to correct, if possible, whatever is amiss in the system at large, or in the state of particular functions.

In cases where there is a weakened, debilitated condition of the system, the employment of tonics will be found quite valuable; of these the carbonate of iron has generally been looked upon almost as a specific; and the beneficial effects of this remedy is supposed to be owing to its giving firmness to the nervous system, by increasing the quantity of red blood that circulates in it. Combined with the iron, local applications to the affected part, of belladonna, and aconite, have been tried with advantage. The internal exhibition of narcoties may also be demanded where there is little or no disposition to sleep on retiring to bed. In cases of an intermittent character dependent upon malarious influences, sulphate of quinia or Fowler's solution will be found most reliable.

When every thing has been done toward improving the general health without affording any relief to the local difficulty, recourse may be had to topical expedients, such as division of the trunk of the painful nerve, if possible, between the seat of irritation and the brain. With this object in view, many years ago, division of the infra-orbital nerve, as it emerges from the foramen, was advocated and practiced to a considerable extent. The relief afforded, however, appears to have been very slight and transitory, and in the majority of cases there was almost invariably a return of the suffering.

In the case of Dr. Pemberton, whose sufferings are described as of the most aggravated character, division of all the branches was made by Sir Astley Cooper, but without avail. This practice, though still pursued, has been to a great extent abandoned, from a conviction that the seat of the difficulty is not reached by it.

Within the past four years, Dr. Carnochan has advocated exsection of the trunk of the second branch of the fifth pair, beyond the ganglion of Meckel, removing at the same time the ganglion, or insulating it and its branches from the encephalon. This operation, which he has performed on three different occasions, is quite a formidable one, and when the cause of suffering is posterior to the ganglion, will prove as unavailing as section of the peripheral extremity of the nerve. The operation, as performed by him, is as follows: The integuments of the cheek are dissected away from the superior maxilla so as to completely expose the anterior wall of the antrum. The anterior and posterior walls of the antrum are then perforated by means of the trephine and chisels By this means an opening having been made into the spheno-maxillary fossa, the ganglion of Meckel is readily reached and removed. A portion of the inferior maxillary nerve, about two inches in length, is removed at the same time. Dr. Carnochan states that in the three cases performed by him, the relief afforded by the operation has continued from that period.

Excision of the inferior dental branch of the lower maxillary nerve has also been frequently performed in cases where the pain has been confined to the lower jaw, affording, according to the statement of operators, prompt and enduring relief. In performing this operation, an incision is first made through the integuments over the ramus of the lower jaw. The nerve is then exposed by applying the crown of a trephine upon the bone at a point immediately below the entrance of the nerve into the dental canal.—*Dental Cosmos.*

